# Measurement Invariance of the Sexual Orientation Microaggression Inventory across LGB Males and Females in Taiwan: Bifactor Structure Fits the Best

**DOI:** 10.3390/ijerph182010668

**Published:** 2021-10-12

**Authors:** Meng-Tsang Hsieh, Jung-Sheng Chen, Chung-Ying Lin, Cheng-Fang Yen, Mark D. Griffiths, Yu-Te Huang

**Affiliations:** 1 Stroke Center and Department of Neurology, E-Da Hospital, Kaohsiung 82445, Taiwan; hmtspider@gmail.com; 2 School of Medicine for International Students, College of Medicine, I-Shou University, Kaohsiung 82445, Taiwan; 3 Institute of Clinical Medicine, College of Medicine, National Cheng Kung University, Tainan 70101, Taiwan; 4 Department of Medical Research, E-Da Hospital, Kaohsiung 82445, Taiwan; ed113187@edah.org.tw; 5 Institute of Allied Health Sciences, College of Medicine, National Cheng Kung University, Tainan 70101, Taiwan; 6 Department of Occupational Therapy, College of Medicine, National Cheng Kung University, Tainan 70101, Taiwan; 7 Department of Public Health, National Cheng Kung University Hospital, College of Medicine, National Cheng Kung University, Tainan 70101, Taiwan; 8 Biostatistics Consulting Center, National Cheng Kung University Hospital, College of Medicine, National Cheng Kung University, Tainan 701, Taiwan; 9 Department of Psychiatry, School of Medicine College of Medicine, Kaohsiung Medical University, Kaohsiung 80708, Taiwan; 10 Department of Psychiatry, Kaohsiung Medical University Hospital, Kaohsiung 80756, Taiwan; 11 College of Professional Studies, National Pingtung University of Science and Technology, Pingtung 91201, Taiwan; 12 International Gaming Research Unit, Psychology Department, Nottingham Trent University, Nottingham NG1 4FQ, UK; mark.griffiths@ntu.ac.uk; 13 Department of Social Work and Social Administration, The University of Hong Kong, Hong Kong RM543, China; Yuhuang@hku.hk

**Keywords:** factor analysis, microaggression, sexual orientation, lesbian, gay, bisexual, mental health, Taiwan

## Abstract

The present study examined the factor structure and concurrent validity of the traditional Chinese version of the Sexual Orientation Microaggression Inventory (SOMI) among lesbian, gay, and bisexual (LGB) individuals in Taiwan. In total, 1000 self-identified LGB individuals completed the SOMI, HIV and Homosexuality Related Stigma Scale (HHRSS), and Acceptance and Action Questionnaire-II (AAQ). Different factor structures (including one-factor, four-factor, bifactor, and higher-order factor structures) were evaluated using confirmatory factor analysis. The bifactor structure significantly outperformed all others on the SOMI. The bifactor structure with one general factor and four trait factors was found to be measurement invariant across biological sex with satisfactory fit indices. The SOMI general factor was significantly associated with HHRSS-Homosexuality score and AAQ score. The findings indicate that the SOMI is a psychometrically sound instrument for Taiwan sexual minority groups. More specifically, SOMI can be used to accurately assess microaggression among LGB individuals. The measure on microaggression may also provide insights for healthcare providers about LGB individuals’ sexuality-related stigma. Moreover, healthcare providers and relevant stakeholders can use the SOMI to understand how LGB individuals perceive and feel microaggression.

## 1. Introduction

Lesbian, gay, and bisexual (LGB) individuals may experience sexual prejudice in diversified forms, such as verbal and physical bullying, differential treatment, and hate crimes [1,2]. According to minority stress theory [3], sexual prejudice derived from heterosexism increases stress among LGB individuals and compromises their health [4]. However, LGB individuals may not only experience overt acts but also covert acts of sexual prejudice. Microaggression against LGB individuals is a common form of covert aggression that often goes unnoticed [5,6]. ‘Microaggression’ emerged as a term for describing acts of subtle racism, and is defined as “brief and commonplace daily verbal, behavioral, or environmental indignities, whether intentional or unintentional, that communicate hostile, derogatory, or negative racial slights or insults” (p. 72) [7]. Sexual orientation microaggression is further classified into three forms: microassault, microinsult, and microinvalidation [6,7]. Microassaults refer to discriminatory verbal or non-verbal behaviors against LGB individuals rooted in heterosexism (e.g., using the term “that’s so gay” for describing others in a derogatory way). Microinsults refer to “subtle snubs” due to sexual minority identity (e.g., a store clerk ignoring LGB individuals). Microinvalidations refer to the engagement of communications that nullify the stigmatized experiences of a LGB individual (e.g., LGB individuals receiving comments such as “Don’t be so sensitive” when talking about a stigmatized experience) [5,6]. 

Research has shown that among LGB individuals, the experience of microaggression increases the risks of mental health problems such as depression [5], anxiety [5,8], and posttraumatic stress symptoms [9], as well as being associated with low self-acceptance and self-esteem [8,10], and negative feelings toward sexual identity [10]. The experience of microaggression has also been found to predict non-response to psychotherapy among LGB individuals [11]. The results of previous studies indicate that it is important to assess the experience of microaggression and its impact on psychological wellbeing among LGB individuals. Indeed, Asian-Pacific regions with the efforts of United Nations Education Scientific and Cultural Organization (UNESCO) have identified this as an issue for LGB individuals (i.e., developing programs to fight LGB aggression) [12,13]. However, these programs put the most emphasis on school-aged students and there is a literature gap among older populations (e.g., young adults).

A number of robust instruments assessing three forms of microaggression have been developed. For example, the 45-item Homonegative Microaggressions Scale (HMS) was developed with target respondents being primarily White adults [10]. The 18-item LGBT People of Color Microaggressions Scale (LGBT-POC) was developed with the target respondents being diverse minority populations [14]. The 19-item Sexual Orientation Microaggression Inventory (SOMI) was developed using the theoretical framework proposed by Sue et al. [7] and its psychometric properties were tested among a diverse sample of LGBT youth [6]. The present study examined the factor structure of the SOMI rather than the HMS and LGBT-POC among Taiwan LGB individuals for a number of reasons: (i) the HMS (45 items) contains many more items than the SOMI (19 items), and (ii) the content of the LGBT-POC includes more than just LGB-specific microaggression (i.e., the LGBT-POC includes items assessing racism). Therefore, using SOMI to assess microaggression has the benefits of a (i) much shorter administration time, and (ii) being solely focused on LGB-specific microaggression.

Research among two American samples confirmed that the SOMI has a bifactor structure involving a general factor and four specific factors (anti-gay attitudes and expressions, denial of homosexuality, heterosexualism, and societal disapproval) [6]. However, to the best of the present authors’ knowledge, the SOMI has never been examined in other populations other than Americans. Although legislation for same-sex relationships in Taiwan has been introduced [15], anti-LGB stigma is prevalent in Taiwan. 

The Gender Equality Education Act was implemented in Taiwan on 25 June 2004 [16]. The law requires schools to provide a gender equitable learning environment, and give respect and due consideration to students, regardless of their gender, gender disposition, gender identity, or sexual orientation [16]. However, research found that while the Gender Equality Education Act results in positive influences on school policy and curricular development, teachers, principals, and administrators often lack necessary knowledge and skills to implement the act [17]. Moreover, anti-LGB groups in Taiwan drafted a referendum that called for a ban on homosexual-related education in elementary and junior high schools. The Taiwanese adult population voted on this referendum on 24 November 2018, and approximately seven million Taiwanese (67.44%) voted in support of this referendum [18]. Although the Ministry of Education in Taiwan insists the necessity of homosexual-related education for students, the promotion of sexual orientation equality in schools is beset with difficulties. Furthermore, there is no anti-discrimination law in Taiwan to protect the right of LGB individuals from violation. There are no formal anti-discrimination policies formulated by the government in Taiwan to enhance the awareness and changes of stigma towards sexual and gender minorities in the public. Consequently, stigmatizing attitudes and behaviors towards LGB individuals are prevalent.

For example, a study on young adult gay and bisexual men found that 56.4% of participants had experienced homophobic bullying during their childhood [19]. Consequently, to assess microaggression and improve LGB health in Taiwan, validating the factor structure of the SOMI among Taiwan LGB individuals is important for healthcare providers and related stakeholders. Emerging adulthood is a phase of the lifespan from adolescence to full adulthood where individuals become more independent and explore various life possibilities [20]. Several psychiatric disorders including anxiety disorders, mood disorders, impulse control disorders, and substance use disorders typically begin during emerging adulthood [21,22]. Consequently, to assess microaggression and improve LGB health among young adult LGB individuals in Taiwan, validating the factor structure of the SOMI among Taiwan LGB individuals during young adulthood is important for healthcare providers and related stakeholders.

More specifically, the present study rigorously examined the factor structure of the SOMI among Taiwan LGB individuals for two main purposes. First, to examine whether the SOMI has the same bifactor structure among Taiwan LGB individuals as found among Americans. Second, to examine whether the SOMI’s factor structure is measurement invariant across biological sex among LGB individuals. For the first purpose, several potential factor structures (including a one-factor structure, a four-factor structure, a bifactor structure, and a higher-order factor structure) were tested to verify the best fitting factor structure for the SOMI. For the second purpose, multigroup analyses were carried out to examine whether the best-fitting model found is measurement invariant across sex.

## 2. Materials and Methods

### 2.1. Participants and Procedure

The participant inclusion criteria were individuals who identified their sexual orientation as being homosexual or bisexual, aged between 20 and 30 years, and living in Taiwan. Participants were recruited by posting an online advertisement on social media including *Facebook, Twitter,* and *LINE* (a direct messaging app), the Bulletin Board System, and the home pages of three health promotion and counseling centers for LGB individuals from August 2018 to July 2020. Anyone who intended to participate in the study telephoned the research assistants. The research assistant ensured the eligibility of potential participants for recruitment criteria, explained the study aims and procedures to them, and scheduled the time for completing the study questionnaires individually in the study room. The research assistants evaluated the participants in the on-site study room to determine whether they had impaired intellect or showed signs of alcohol and substance use that might interfere with understanding the study’s purpose or to complete the questionnaire. In total, 1000 participants (500 males and 500 females) participated in the study. No participants were excluded. Informed consent was obtained from all participants prior to the assessment. The study was approved by the Institutional Review Board of Kaohsiung Medical University Hospital (KMUHIRB-F(II)-20180018).

### 2.2. Measures

#### 2.2.1. Sexual Orientation Microaggression Inventory (SOMI) and Its Chinese Translation

The SOMI comprises 19 items assessing microaggression among LGB individuals with four trait factors, including anti-gay attitudes and expressions (six items), denial of homosexuality (three items), heterosexualism (five items), and societal disapproval (five items) [6]. The SOMI items are rated on a five-point scale from 1 (not at all) to 5 (almost every day). Therefore, a higher SOMI score indicates a higher level of microaggression. The psychometric properties of the SOMI were found to be satisfactory in prior research. For example, the criterion-related validity of the SOMI was satisfactory (r = 0.65 with concurrent victimization measure; 0.53 with six-month later victimization measure; 0.25 with concurrent internalizing measure, and 0.21 with six-month later internalizing measure) [6]. Moreover, the SOMI was found to have a bifactor structure in psychometric testing. More specifically, there is a general factor in the SOMI apart from the aforementioned four trait factors. The internal consistency of the SOMI in the present sample was good to excellent (Cronbach’s α = 0.74 [anti-gay attitudes and expressions], 0.72 [denial of homosexuality], 0.78 [heterosexism], 0.85 [societal disapproval], and 0.90 [entire SOMI]).

The SOMI was translated into the traditional Chinese version for Taiwanese LGB individuals using the standard forward-, backward-, and pretest-step methods [23]. First, the original version was translated into the traditional Chinese version by one bilingual translator. Next, the traditional Chinese version was back-translated into English by another bilingual translator. Finally, the original version was compared with the back-translation. If discrepancies arose in the back-translation, translators worked cooperatively to make corrections in the final traditional Chinese version. Three further experts in the field of sexuality study were invited to examine the adequacy of the translated scale.

#### 2.2.2. Measures Used for Concurrent Validity of the SOMI

Two measures for examining the concurrent validity of the SOMI were used. The first measure used was the HIV and Homosexuality Related Stigma Scale (HHRSS) [24]. The study adopted the 12 items of the HHRSS-Homosexuality assessing stigma attitudes towards homosexuality that LGB individuals perceive from their families. The items are rated on a four-point scale from 1 (strongly disagree) to 4 (strongly agree). Therefore, a higher HHRSS-Homosexuality score indicates a higher level of perceived stigma on homosexuality. Prior research has found the psychometric properties of the HHRSS-Homosexuality to be satisfactory [24]. Additionally, the HHRSS-Homosexuality internal consistency in the present sample was excellent (Cronbach’s α = 0.93).

The second measure used was the Acceptance and Action Questionnaire-II (AAQ) [25]. The AAQ comprises seven items assessing an individual’s psychological flexibility. All the AAQ items are rated on a seven-point scale from 1 (completely disagree) to 7 (completely agree). Therefore, a higher AAQ score indicates a lower level of psychological flexibility. Prior research has found the psychometric properties of the AAQ to be satisfactory [25,26,27]. Additionally, the AAQ internal consistency in the present sample was excellent (Cronbach’s α = 0.92).

### 2.3. Data Analysis

Before testing the factor structure and the measurement invariance of the SOMI, the participants’ characteristics and the SOMI item scores were analyzed via descriptive statistics. Moreover, SOMI item distributions were assessed with acceptable skewness in absolute values at 3 or below, and acceptable kurtosis in absolute values at 10 or below [28]. When testing the factor structure of the SOMI, confirmatory factor analysis (CFA) was used to examine four different models: Model 1 is a one-factor structure model; Model 2 is a four-factor structure model; Model 3 is a bifactor model with four trait factors and one general factor, and Model 4 is a higher-order model with one second-order factor and four first-order factors. All the CFA models were performed using a diagonally weighted least squares estimator to account for the categorical responses in the SOMI. Additionally, a set of fit indices was used to evaluate the four CFA models: both comparative fit index (CFI) and Tucker-Lewis index (TLI) >0.95, root mean square error of approximation (RMSEA) <0.06, and standardized root mean square residual (SRMR) <0.08 [29]. If all four models have satisfactory fit indices, a χ^2^ difference test is used to examine whether any proposed model has a significantly better fit than the other models.

When the best-fitting model was determined, the following testing in measurement invariance across and concurrent validity was assessed using the best-fitting model. In the measurement invariance, three nested models were proposed: Model a is a configural model that freely estimates both factor loadings and item intercepts across sex; Model b is a model that constrains the factor loadings being equal across sex; Model c is a model that constrains both factor loadings and item intercepts being equal across sex. In order to assess whether the measurement invariance of the SOMI is supported, χ^2^ difference test, ΔCFI, ΔRMSEA, and ΔSRMR were used. More specifically, a nonsignificant χ^2^ difference test suggests measurement invariance. However, given that the χ^2^ difference test can easily be significant in a large sample (e.g., *n* = 1000 in the present sample) [30], ΔCFI, ΔRMSEA, and ΔSRMR were supplement indices to determine the invariance. According to Chen [31], the ΔCFI > −0.01, ΔRMSEA < 0.015, together with ΔSRMR < 0.01 indicate that the measurement invariance is supported.

The concurrent validity of the SOMI was examined using the correlations between SOMI factors (including both trait and general factors) and the two external measures of HHRSS-Homosexuality and AAQ. More specifically, it was hypothesized that the SOMI trait and general factors would be positively associated with both HHRSS-Homosexuality and AAQ because: (1) HHRSS-Homosexuality assesses stigma toward homosexuality [24], which is a concept that should be positively associated with the perceptions of microaggression, and (2) AAQ assesses psychological flexibility [25], which is also a concept that should be associated with microaggression. Moreover, the association between AAQ and SOMI was hypothesized to be positive because lower scores on the AAQ indicate higher psychological flexibility, and higher score on the SOMI indicate higher microaggression. The psychometric testing was done using the *lavaan* package in the R software (https://lavaan.ugent.be/index.html, accessed on 1 October 2021) for all the CFA-related evaluations. All the other analyses were done using the IBM SPSS 20.0 (Armonk, NY, USA: IBM Corp).

## 3. Results

The participants included 1000 self-identified LGB individuals (500 males and 500 females), of which the mean age was 24.63 years (SD = 2.99). The total sample had a good education (89.1% had a college or above degree), and more than half of them were homosexual (57.0%) with the rest of the participants identifying as bisexual. More than half of the participants had their sexual orientation known by friends (64.6%) or online friends (52.4%). However, less than a quarter of the participants had their sexual orientation known by their family (21.9%). Detailed information on the participants’ characteristics is reported in Table 1.

All the 19 items in the SOMI were, in general, normally distributed (skewness = 0.14 to 1.58; kurtosis = 0.06 to 2.41) with their mean (SD) ranged between 1.61 and 3.33 (0.81 and 1.13; Table 2). Moreover, all the tested CFA models had satisfactory fit in terms of CFI (0.956 to 0.998), TLI (0.951 to 0.997), RMSEA (0.017 to 0.065), and SRMR (0.035 to 0.081); with the exception of significant χ^2^ test (all *p*-values <0.001). χ^2^ difference tests further showed that the bifactor model (i.e., Model 3 in Table 3) significantly outperformed other models (Δχ^2^ [Δdf] = 629.45 [25] vs. Model 1 [i.e., one-factor structure factor]; = 276.56 [19] vs. Model 2 [i.e., four-factor structure factor]; = 289.39 [21] vs. Model 4 [i.e., higher-order structure with one second-order factor and four first-order factors]; all *p*-values <0.001). Indeed, all the fit indices in Model 3 were the best among all the models. Therefore, two types of factor loadings (trait factor loading and general factor loading) were further scrutinized in the bifactor model. The factor loadings are presented in Table 2 with all loadings were significant, except for Item 3 in the anti-gay attitudes and expressions trait (loading = −0.032) and Item 2 in the heterosexism trait (loading = −0.024).

The bifactor structure of the SOMI was additionally examined for its measurement invariance across sexes. The configural model of the SOMI (i.e., Model 3a in Table 4) had excellent fit in all the fit indices, including the nonsignificant χ^2^ test (*p*-value = 0.964). The model constrained factor loadings equally across sexes (i.e., Model 3b), and the model constrained both factor loadings and item intercepts across sexes (Model 3c) had significantly worse fit than the configural model in terms of χ^2^ difference tests. However, other fit indices in measurement invariance all indicated that the bifactor model was invariant across sex (ΔCFI = −0.003 and −0.002; ΔRMSEA = 0.008 and 0.015; ΔSRMR = 0.003 and 0.009).

Finally, the bifactor structure was used to examine the concurrent validity of the SOMI with HHRSS-Homosexuality and AAQ. Correlation coefficients showed that almost all the traits and the general factor of the SOMI were significantly and positively associated with HHRSS-Homosexuality (r = 0.120 to 0.336; *p*-values < 0.05) and AAQ (r = 0.090 to 0.262; *p*-values < 0.01; Table 5).

## 4. Discussion

The present findings corroborate the bifactor structure of the SOMI found by Swann et al. [6] and extended the bifactor structure to an East-Asian population who experienced the legalization of same-sex relationship (i.e., Taiwanese LGB individuals). This is an important topic given that the present findings echo prior findings from Asia-Pacific research [32] that the denial of homosexuality is a core precept in religious conversion of LGB individuals. Consequently, LGB individuals encounter health issues such as high levels of suicidality, physical abuse, and homelessness. Moreover, the present findings share the same findings with Swann et al. [6] that two items (i.e., SOMI Item 3 in anti-gay attitudes and expression, and SOMI Item 2 in heterosexualism) were not significant in their embedded trait factors. In the bifactor structure, the four trait factors corresponded well with the three microaggression forms proposed by Sue et al. [7]. More specifically, microassaults share similar meaning with the trait factor of societal disapproval, microinsults with the trait factor of anti-gay attitudes, and microinvalidation with the trait factor of heterosexism; while the trait factor of denial of homosexuality is a new and unique concept to LGB individuals [6]. Moreover, the present findings supplement the present literature by showing that the bifactor structure of the SOMI is measurement invariant across biological sex, which indicates the appropriateness to use the SOMI to compare different levels of microaggression between sexes. The concurrent validity of the SOMI was somewhat supported by the HHRSS-Homosexuality and AAQ, which also justifies that the use of SOMI is appropriate.

The bifactor structure of the SOMI may cause challenges in interpreting both trait and general factors simultaneously [33]. Nevertheless, a solution has been proposed by Swann et al. [6]. More specifically, they recommend using the SOMI general factor as the primary index to assess general microaggression instead of using SOMI trait factors to assess each form of microaggression. The reasons for focusing on the SOMI general factor include (i) the general factor serves as a superior measure to represent all the items given that all the variances of each specific item characteristics are controlled for [34,35,36], and (ii) the different forms of microaggression are likely to overlap with each other and result in the difficulty of distinctly and separately assessing them [37]. In brief, with the use of the SOMI general factor, those using the instrument can have an overall assessment of microaggression. Subsequently, the interpretations of microaggression can be simplified and this practice provides straightforward information for healthcare providers or other stakeholders to have clear information whether an LGB individual has a microaggression issue.

The SOMI general factor was found to be associated with homosexuality-related stigma from family and with psychological flexibility. This demonstrates that the SOMI has concurrent validity. More specifically, microaggression is a type of stigma and it should be associated with another type of stigma assessed using the HHRSS-Homosexuality [24]. Moreover, psychological flexibility is an important personal characteristic that assists an individual in coping with unfavored environments [25]. Therefore, microaggression should be associated with psychological flexibility. Moreover, given that microaggression, homosexuality-related stigma, and psychological flexibility are different concepts, their associations should not be strong. Interestingly, the association between SOMI general factor and homosexuality-related stigma from online information was weak and negative. This finding somewhat contradicts what was hypothesized and the possible reason may be that the participants were well educated and were able to ignore the insults posted online. However, more evidence is needed to corroborate such speculation. 

Given that sexual orientation microaggression has negative impacts on mental health among LGB individuals [5,8,9,10], governments are urged to develop intervention programs for reducing microaggression induced by individuals’ views on sexual orientation. However, compared with anti-LGB bullying prevention, anti-LGB microaggression prevention has only just started. According to the UNESCO [12,13], broadening awareness and understanding of microaggression in educational settings, workplaces, and home environments are the necessary step to help overcome the issue. The results of the present study indicated that the SOMI is a psychometrically sound instrument to help raise awareness and identify experiences of microaggression induced by individuals’ views on sexual orientation. Consequently, policies can be developed to reduce such micro-aggressive behavior.

There are some limitations in the present study. First, the present sample comprised well-educated LGB individuals (nearly 90% of the participants had a college degree or above). Therefore, it is unclear whether the SOMI would maintain its factor structure in populations with a lower level of education. Similarly, the present sample did not include any aboriginals, and the generalizability of the present study’s findings is restricted. Future studies using participants with diverse backgrounds are needed to confirm the bifactor structure of the SOMI. Second, some important psychometric properties (including test-retest reliability and responsiveness) were not examined in the present study. Without the information of test-retest reliability, it is hard to conclude that the SOMI is stable in capturing microaggression across time [38]. Without information concerning responsiveness, it cannot be ensured that the SOMI can capture changes of microaggression among LGB individuals [39]. Consequently, it will be somewhat difficult to use the SOMI to evaluate the effects of a program on microaggression reduction. Third, all the data collected in the present study (i.e., SOMI, HHRSS, and AAQ) were self-reported. Therefore, single-rater biases, recall biases, and social desirability biases cannot be fully controlled [40,41,42]. Future studies using other objective measures to examine the concurrent validity of the SOMI are therefore warranted.

## 5. Conclusions

The present study demonstrated that the Chinese version of SOMI applied to the Taiwan sexual minority group (i.e., LGB individuals) has satisfactory psychometric properties with regards to its factor structure and its internal consistency. The bifactor structure was confirmed in the sample and was found to be invariant across males and females. In addition, the concurrent validity of the SOMI was somewhat supported by other relevant instruments, including the HHRSS-Homosexuality and AAQ scales. Given that the psychometric properties of the SOMI were supported in the present study, healthcare providers and relevant stakeholders may want to use the SOMI to understand how LGB individuals perceive and feel microaggression (including microassault, microinsult, and microinvalidation) in Taiwanese community and society. With enhanced information concerning microaggression, subsequent actions may be taken to improve the LGB individuals’ health and living environments. More specifically, policies concerning anti-homophobic education for students have been proposed by the UNESCO. Therefore, the SOMI can be used to assess microaggression and help policymakers in developing effective anti-homophobic education.

## Figures and Tables

**Table 1:** Characteristics of the participants (*n* = 1000).

	*n* (%)
Age in year ^a^	24.63 (2.99)/20–30
Educational level	
High school or below	109 (10.9)
College or above	891 (89.1)
Sex	
*Male*	500 (50.0)
*Female*	500 (50.0)
Sexual orientation	
*Homosexual*	570 (57.0)
*Bisexual*	430 (43.0)
Father’s education	
*High school or below*	591 (59.1)
*College or above*	409 (40.9)
Mother’s education	
*High school or below*	660 (66.0)
*College or above*	340 (34.0)
Sexual orientation known by family	
*None or few*	781 (78.1)
*Many or a great quantity*	219 (21.9)
Sexual orientation known by friends	
*None or few*	354 (35.4)
*Many or a great quantity*	646 (64.6)
Sexual orientation known by online friends	
*None or few*	476 (47.6)
*Many or a great quantity*	524 (52.4)

^a^ Reported mean (SD)/range.

**Table 2:** Item properties of the Sexual Orientation Microaggression Inventory (SOMI).

	Trait Factor Loading	General Factor Loading	Mean (SD)	Skewness	Kurtosis
**A**					
SOMI1	0.227	0.536	2.18 (0.91)	0.78	0.51
SOMI3	**−0.032**	0.640	1.61 (0.81)	1.42	2.02
SOMI6	0.332	0.373	2.08 (1.05)	0.82	0.06
SOMI7	0.276	0.421	1.75 (1.00)	1.34	1.22
SOMI8	0.656	0.394	2.86 (1.04)	0.19	−0.51
SOMI9	0.568	0.415	2.34 (1.09)	0.49	−0.52
**D**					
SOMI4	0.587	0.440	1.74 (0.87)	1.24	1.50
SOMI5	0.777	0.475	1.76 (0.86)	1.24	1.59
SOMI14	0.242	0.456	1.95 (1.08)	1.19	0.87
**H**					
SOMI2	**−0.024**	0.639	1.60 (0.86)	1.58	2.41
SOMI16	0.401	0.537	1.72 (0.92)	1.23	0.92
SOMI17	0.635	0.332	2.27 (1.12)	0.67	−0.31
SOMI18	0.476	0.549	1.85 (1.02)	1.17	0.70
SOMI19	0.826	0.288	2.44 (1.11)	0.41	−0.59
**S**					
SOMI10	0.638	0.276	3.33 (1.08)	−0.14	−0.72
SOMI11	0.702	0.277	2.67 (1.13)	0.32	−0.69
SOMI12	0.671	0.357	2.69 (1.09)	0.26	−0.56
SOMI13	0.571	0.433	2.60 (1.09)	0.45	−0.42
SOMI15	0.647	0.399	2.57 (1.11)	0.40	−0.56

A = anti-gay attitudes and expressions; D = denial of homosexuality; H = heterosexism; S = societal disapproval; G = general factor. Nonsignificant values are in bold. Factor loadings were derived from the bifactor model of the SOMI in the confirmatory factor analysis.

**Table 3:** Model comparisons for the Sexual Orientation Microaggression Inventory (SOMI).

	Fit Statistics			
Model	Model 1	Model 2	Model 3	Model 4
χ^2^ (df)	792.19 (152)	439.30 (146)	162.74 (127)	452.14 (148)
*p*-value	<0.001	<0.001	0.018	<0.001
CFI	0.956	0.980	0.998	0.979
TLI	0.951	0.976	0.997	0.976
RMSEA	0.065	0.045	0.017	0.045
90% CI of RMSEA	0.061, 0.069	0.040, 0.050	0.007, 0.024	0.041, 0.050
SRMR	0.081	0.060	0.035	0.061
**Comparison**	**Model 1 vs. 3**	**Model 2 vs. 3**	**--**	**Model 4 vs. 3**
Δχ^2^ (Δdf)	629.45 (25)	276.56 (19)	--	289.39 (21)
*p*-value	<0.001	<0.001	--	<0.001

Model 1 = one-factor structure model. Model 2 = four-factor structure model. Model 3 = bifactor model with four trait factors and one general factor. Model 4 = higher-order model with one second-order factor and four first-order factors. CFI = comparative fit index; TLI = Tucker–Lewis index; RMSEA = root mean square error of approximation; SRMR = standardized root mean square residual; CI = confidence interval; Δ = difference.

**Table 4:** Measurement invariance of the bifactor structure for the Sexual Orientation Microaggression Inventory (SOMI) across sex.

		Fit Statistics	
Model	Model 3a	Model 3b	Model 3c
χ^2^ (df)	215.07 (254)	320.85 (287)	377.14 (301)
*p*-value	0.964	0.083	0.002
CFI	1.000	0.998	0.995
TLI	1.004	0.997	0.994
RMSEA	0.000	0.015	0.023
90% CI of RMSEA	0.000, 0.000	0.000, 0.024	0.014, 0.029
SRMR	0.038	0.047	0.050
**Comparison**	**--**	**Model 3a vs. 3b**	**Model 3a vs. 3c**
Δχ^2^ (Δdf)	--	105.78 (33)	56.29 (14)
*p*-value	--	<0.001	<0.001
ΔCFI	--	−0.002	−0.003
ΔRMSEA	--	0.015	0.008
ΔSRMR	--	0.009	0.003

Model 3a = bifactor structure of SOMI in configural model across sex. Model 3b = bifactor structure of SOMI with factor loadings constrained equal across sex. Model 3c = bifactor structure of SOMI with factor loadings and item intercepts constrained equal across sex. CFI = comparative fit index; TLI = Tucker–Lewis index; RMSEA = root mean square error of approximation; SRMR = standardized root mean square residual; CI = confidence interval; Δ = difference.

**Table 5:** Concurrent validity of the Sexual Orientation Microaggression Inventory (SOMI) with HIV and Homosexuality Related Stigma (HHRSS), and Acceptance and Action Questionnaire-II (AAQ).

SOMI Factor	r (*p*-Value)	
	HHRSS-Homosexuality	AAQ
A	0.120 (<0.001)	0.147 (<0.001)
D	0.250 (0.039)	0.090 (0.003)
H	0.201 (<0.001)	0.209 (<0.001)
S	0.251 (<0.001)	0.157 (<0.001)
G	0.336 (<0.001)	0.262 (<0.001)

A = anti-gay attitudes and expressions; D = denial of homosexuality; H = heterosexism; S = societal disapproval; G = general factor.

## Data Availability

The data will be available upon reasonable request to the corresponding authors.
